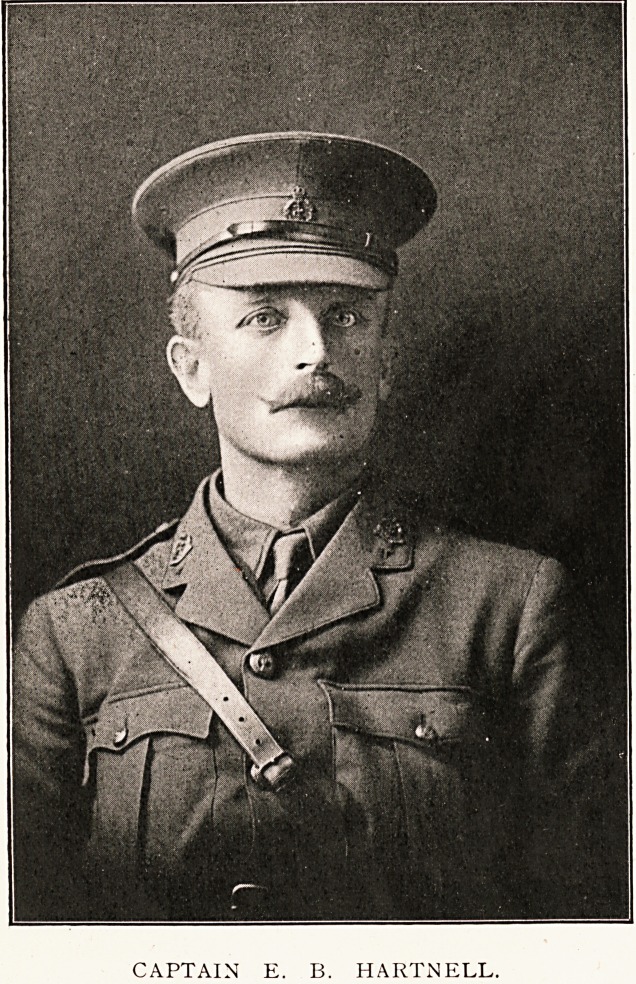# Captain E. B. Hartnell

**Published:** 1916-07

**Authors:** 


CAPTAIN E. B. HARTNELL.

### Obituary.

### CAPTAIN E. B. HARTNELL.

We deeply regret to have to record the death at Cairo of Captain Edward Bush Hartnell, in his forty-ninth year, the result of dysentery with acute appendicitis, contracted while on active service. His father, the late Rev. Bedford Hartnell, was for many years a House Master at Clifton College, and for some time the Head Master of the Junior School there. Edward Hartnell entered the Bristol Medical School, and towards the end of his student career was at Guy's Hospital. He obtained the qualifications of M.R.C.S. and L.R.C.P. in 1892, and shortly afterwards became assistant to the late Dr. E. M. Grace, of Thornbury, with whom he was associated for four years. He married Dr. Grace's daughter Alice. Leaving Dr. Grace, he set up in practice in Bridgwater in 1908.

Edward Hartnell was a keen sportsman, and responded early to the call for practitioners to join the R.A.M.C., for on September 21st, 1914, within a month of the outbreak of hostilities, he became attached to the London Mounted Brigade Field Ambulance. He displayed aptitude and enthusiasm for his new work, and very shortly afterwards was raised to the rank of Captain. With a brief period of training at North Walsham, he sailed from Avonmouth for Egypt, and for a short time was placed in charge of the Anglo-American Hospital. Subsequently he was transferred to Gallipoli, where he saw a good many severe engagements, and performed valuable work for the wounded there. In December, 1915, he returned to Egypt, and after working in various camps, he proceeded to the Suez Canal, where he remained until he was attacked by dysentery, and transferred to the Nazrich Hospital, Cairo. Here appendicitis developed, and, weakened by his previous illness, he succumbed on April 25th.

Edward Hartnell had earned the warm regard of his colleagues at Bridgwater, and was held in very high esteem by his many patients and friends there, by whom the loss of so sure and good a friend is as sincerely mourned as amongst his old associates and his teachers and fellow-students at Bristol and Guy's.

We quote a short abstract from the reference made by the Vicar of St. Mary's, Bridgwater, the Rev. J. J. Langham, at the service following news of his death :—

" By the death of Captain E. B. Hartnell, while engaged in the service of his country, this church and parish has lost a loyal and devoted worker. It was indeed with a distinct sense of loss that we heard that at the beginning of the war he had obeyed the call of King and Country. We wondered how we should get on without him, and were looking forward to the time when he would be able to return at the end of the war and resume his activities. But God has willed it otherwise, and has called him to the higher service in Paradise. Meanwhile he has left behind him a rich legacy of example—the example of a life of devoted service to his God and his church.

"We pray that God will give us grace so to follow his good example, that, with him, we may be partakers of God's heavenly kingdom."

Captain Hartnell leaves a widow, one daughter aged 12, and a son aged 9. To them we extend our deepest sympathy.

## Figures and Tables

**Figure f1:**